# Tuning Transpiration by Interfacial Solar Absorber‐Leaf Engineering

**DOI:** 10.1002/advs.201700497

**Published:** 2017-12-02

**Authors:** Shendong Zhuang, Lin Zhou, Weichao Xu, Ning Xu, Xiaozhen Hu, Xiuqiang Li, Guangxin Lv, Qinghui Zheng, Shining Zhu, Zhenlin Wang, Jia Zhu

**Affiliations:** ^1^ National Laboratory of Solid State Microstructures College of Engineering and Applied Sciences School of Physics, and Collaborative Innovation Center of Advanced Microstructures Nanjing University Nanjing 210093 P. R. China

**Keywords:** interfacial, leaf, solar absorber, transpiration, vapor

## Abstract

Plant transpiration, a process of water movement through a plant and its evaporation from aerial parts especially leaves, consumes a large component of the total continental precipitation (≈48%) and significantly influences global water distribution and climate. To date, various chemical and/or biological explorations have been made to tune the transpiration but with uncertain environmental risks. In recent years, interfacial solar steam/vapor generation is attracting a lot of attention for achieving high energy transfer efficiency. Various optical and thermal designs at the solar absorber–water interface for potential applications in water purification, seawater desalination, and power generation appear. In this work, the concept of interfacial solar vapor generation is extended to tunable plant transpiration by showing for the first time that the transpiration efficiency can also be enhanced or suppressed through engineering the solar absorber–leaf interface. By tuning the solar absorption of membrane in direct touch with green leaf, surface temperature of green leaf will change accordingly because of photothermal effect, thus the transpiration efficiency as well as temperature and relative humidity in the surrounding environment will be tuned. This tunable transpiration by interfacial absorber‐leaf engineering can open an alternative avenue to regulate local atmospheric temperature, humidity, and eventually hydrologic cycle.

Water cycle, namely, hydrologic cycle, mainly consists of continental precipitation, canopy interception, plant transpiration, soil evaporation, and surface streamflow.^1^, ^2^ Plant transpiration, a process of water movement through a plant and its evaporation from aerial parts especially leaves,^3^ consumes a large component of the total continental precipitation (≈48%)^1^, ^2^ and significantly influences global water distribution and climate.^4^ To date, various chemical and/or biological explorations have been made to suppress the transpiration but with uncertain environmental risks.^5^, ^6^, ^7^, ^8^, ^9^ In recent years, interfacial solar steam/vapor generation is attracting a lot of attention for achieving high energy transfer efficiency. And various optical and thermal designs at the solar absorber–water interface for potential applications in water purification,^10^, ^11^, ^12^, ^13^, ^14^, ^15^, ^16^, ^17^, ^18^, ^19^, ^20^, ^21^, ^22^, ^23^ seawater desalination,^24^, ^25^, ^26^, ^27^ and power generation^28^, ^29^ appear.

In this work, we extend the concept of interfacial solar vapor generation to tunable plant transpiration by showing for the first time that the transpiration efficiency can also be enhanced or suppressed through engineering the solar absorber–leaf interface. By tuning the solar absorption of membrane in direct touch with green leaf, surface temperature of green leaf will change accordingly because of photothermal effect, thus the transpiration efficiency as well as temperature and relative humidity in the surrounding environment will be tuned as well. This tunable transpiration by interfacial absorber‐leaf engineering can open an alternative avenue to regulate global atmospheric temperature, humidity, precipitation, and eventually hydrologic cycle.^30^

Transpiration is the process that water is taken up by plant and evaporated from aerial parts like leaves.^3^ Water in the mesophyll cells of leaves evaporates into air through the stomata on the underside of the leaves by photosynthesis or respiration process as follows^31^, ^32^

(1) $$\mathrm{Transpiration}:\;\mathrm{Mesophyll}\;\mathrm{cells}\;\overset{\mathrm{Photosynthesis/Respiration}}{\to }\;H_{2}O↑$$

(2) $$\begin{array}{l} \mathrm{Photosynthesis}:\\ \;\;\;6{\mathrm{CO}}_{2}\;+\;12H_{2}O\;\overset{\mathrm{Light}}{\underset{\mathrm{Chlorophyll}}{\to }}\;C_{6}H_{\mathrm{12}}O_{6}\;+\;6O_{2}↑+\;6H_{2}O↑ \end{array}$$

(3) $$\mathrm{Respiration}:C_{6}H_{\mathrm{12}}O_{6}\;+\;6O_{2}\;+\;6H_{2}O\;\overset{\mathrm{Enzyme}}{\to }\;6{\mathrm{CO}}_{2}↑\;+\;12H_{2}O↑$$

The opening/closure of the stomata bordered by guard cells is dominated by the reducing/increasing water potential of guard cells induced by enhancement/suppression of photosynthesis or respiration.^33^ Therefore, plant transpiration, controlled by the activities of photosynthesis or respiration, can be significantly influenced by the light absorption and leaf temperatures.

As shown in **Figure** **1**a, when the green leaf is coated with opaque white absorber, light absorption is suppressed with enhanced reflection. It is expected that the temperature of the leaf decreases. Consequently, the respiration of the leaf is weakened so that the mesophyll cells are in turgor with a slower vapor output, leading to suppressed transpiration (Figure 1a). Note that, to retain the photosynthesis of the plant (plant survival), the translucent white absorber is preferred. Hence, the transpiration may mainly result from photosynthesis of the green leaf that absorbs the light through translucent white absorber, besides the respiration effect. When the green leaf is coated with black absorber, light absorption is enhanced (Figure 1b), the increased surface temperature of absorber can lead to increased temperature of mesophyll cells and the enhanced transpiration due to the self‐cooling respiration effect of the leaf.^34^, ^35^ With increased water loss due to enhanced transpiration, the cells in the leaf are shriveled in volume. Meanwhile, the water supply (water uptake) of the plant will automatically increase to balance the osmotic pressure between the cells in the leaf and the air.^3^, ^9^

**Figure 1:**
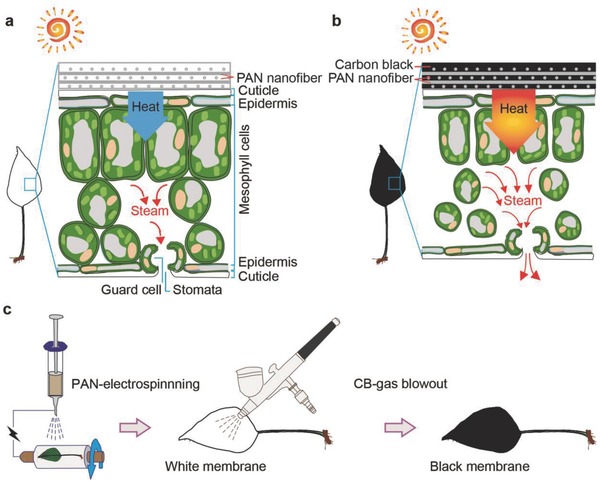
Schematics of tunable transpiration. Transpiration of green leaf coated with a) white absorber and b) black absorber, respectively. c) Fabrication process for engineering absorber–leaf interface.

It should also be noted that tunable transpiration by engineering the absorber–leaf interface is different from natural plant transpiration. For the natural plant transpiration, during daytime with light illumination, transpiration process is accompanied by the photosynthesis. During the nighttime, plant transpiration comes from the respiration effect.^31^ However, for the absorber–leaf interface, the direct light absorption of green leaf is significantly suppressed, so that the tunable transpiration should mainly rely on the respiration effect.

In order to demonstrate the absorption dominated transpiration tunability, two kinds of membranes are directly fabricated on the top surface of green leaf of *Scindapsus aureus* (SA, a complete green plant) via sequential electrospinning process of a polyacrylonitrile (PAN) layer and rapid gas blowout process of a carbon black (CB) layer (≈10 s) (Figure 1c, see the Experimental Section for details).

Due to improved reflectance of light, the white PAN membrane (**Figure** **2**a) is used to suppress transpiration of green leaf (Figure 2b). On the contrary, the black PAN/CB membrane (Figure 2c) is aiming at improving the transpiration because of enhanced light absorption. The white membrane is composed of the PAN nanofiber (average diameter ≈200 nm), and the size of pores formed by the cross‐linked nanofibers is ≈1 µm, as shown in Figure 2d. The black membrane consists of the PAN nanofibers and the loaded CB nanoparticles (see Figure S1, Supporting Information) with diameter of tens of nanometers, as shown in Figure 2e. The white membrane turns to be completely black after the CB deposition, which can be attributed to the sp^2^ orbital hybridization and thus induced π‐π electron transition of the carbon materials.^36^, ^37^ Note that, both the white and black membranes are tightly attached to the leaf surface (i.e., cuticle). The corresponding absorber–leaf interfaces (i.e., PAN‐cuticle and PAN/CB‐cuticle) can be identified by the cross‐sectional scanning electron microscopy (SEM) images, respectively (see Figure 2f,g, and Experimental Section).

**Figure 2:**
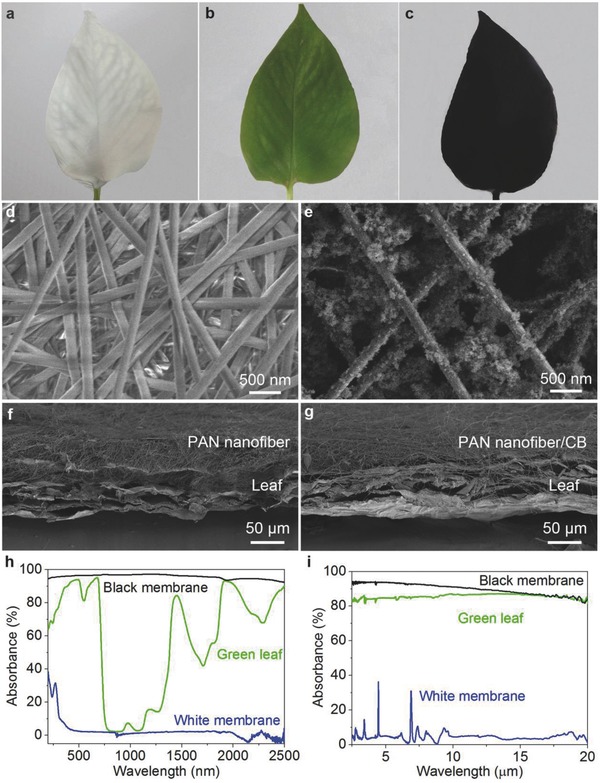
Tunable light absorption properties of the absorbers. Optical photographs of a) the green leaf coated with white membrane, b) green leaf, and c) green leaf coated with black membrane, respectively. Top‐view SEM images of d) white membrane and e) black membrane, respectively. Cross‐sectional SEM images of f) white membrane–leaf interface and g) black membrane–leaf interface, respectively. Experimental absorption spectra measured by h) UV–vis–NIR and i) FT‐IR spectroscopy equipped with the integrated sphere, respectively.

To quantitatively characterize the absorption performance, the absorption spectra of three absorbers (the white PAN membrane, green leaf, and the black PAN/CB membrane) are measured in the wavelength range of 200 nm to 20 µm (Figure 2h,i). Note that in order to ensure the total absorption detection, integrated spheres are equipped in both the ultraviolet–visible–near‐infrared (UV–vis–NIR) and Fourier transform infrared (FT‐IR) spectroscopy measurements (see the Experimental Section). As shown in Figure 2h, the light absorbance of the white membrane is much lower than that of the green leaf, while the absorbance of the black membrane is much higher than that of green leaf. In the UV–vis–NIR regime, the average absorbance of the absorbers weighted by the air mass 1.5 global tilt solar spectrum (AM 1.5 G) reaches ≈97% for the black membrane (≈55% for the green leaf and ≈2% for the white membrane) (Figure S2, Supporting Information). The average absorbance of the green leaf in the mid‐infrared (MIR) regime is about 85% (Figure 2i). The absorption spectrum of white membrane (Figure 2i) shows two dominant absorption peaks at wavelength (λ) = 4.5 µm (2243 cm^−1^) and 6.9 µm (1454 cm^−1^), which can be attributed to the C≡N stretching and CH flat aliphatic vibrations of PAN^38^, ^39^ (Figure S3, Supporting Information). The average absorbance of the white PAN membrane is ≈5% over the MIR regime. After the CB deposition on the PAN membrane, the absorbance of the membrane (black membrane, PAN/CB, Figure 2i) is significantly enhanced. The black membrane shows an averaged absorbance above 90% over the entire MIR regime. Therefore, it is confirmed that the white membrane (the PAN membrane) is beneficial for broadband reflection, while the black membrane (the PAN/CB membrane) is efficient for broadband solar absorption.

To evaluate the tunable transpiration performance, the standard interfacial solar vapor generation experiments were performed^10^, ^12^, ^24^ (see the Experimental Section for details). Transpiration‐induced mass changes as a function of time for the three samples are recorded with dark transpiration totally subtracted, as shown in **Figure** **3**a. In analogy to the interfacial solar vapor generation process, the transpiration efficiency (η) of plant can be defined as^10^, ^15^

(4) $$\eta_{\mathrm{Transp}}=\frac{\dot{m}h_{\mathrm{LV}}}{q_{\mathrm{solar}}}$$

where $\dot{m}$ is the transpiration rate, *h*_LV_ is the latent enthalpy of the liquid–vapor phase change, and *q*_solar_ is the solar irradiation (≈300 W m^−2^). Figure 3b shows the measured transpiration rate ($\dot{m}$) and efficiency (η_Transp_) for three states of the same one leaf. By coating with both membranes, the transpiration performances have been distinctly tuned. In details, the leaf/PAN gives an extremely low transpiration efficiency of ≈8.6% (>70% decrement relative to naked SA green leaf). However, the leaf/PAN/CB enables a pronounced transpiration efficiency of ≈36.4% (nearly 25% enhancement relative to naked SA green leaf, Table S1, Supporting Information). The relative humidity (RH) of the local environment nearby the leaves is tuned accordingly and could be increased with the absorbance of the absorbers (Figure S4, Supporting Information). Note that, as a typical ombrophyte,^40^ the referenced transpiration efficiency of SA (≈29.3% for SA) is higher than most of plants (≈0.1–27.7%) (Table S2, Supporting Information). In addition, ombrophytes commonly suffer from moisture loss due to poor surface coatings.^41^ Therefore, even higher transpiration and humidity tunability in the actual circumstances can be suggested.

**Figure 3:**
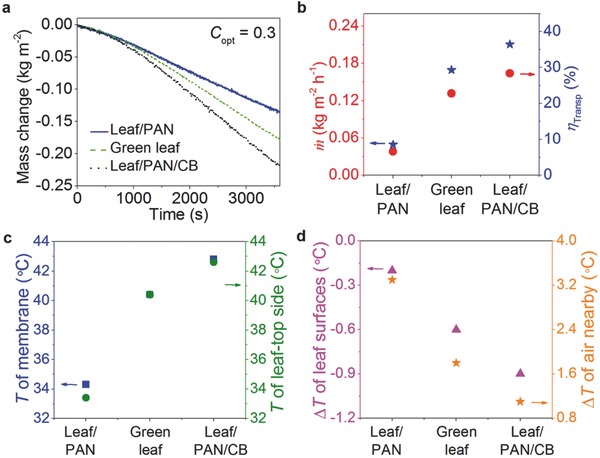
Tunable plant transpiration. a) Transpiration mass over time under 0.3 sun irradiation (*C*_opt_ = 0.3) with dark transpiration subtracted. b) Transpiration rate ($\dot{m}$) and transpiration efficiency (η_Transp_) of green leaf coated with PAN membrane and PAN/CB membrane. c) Temperature (*T*) of the topside of the membrane, temperature of the topside of the green leaf, and d) temperature decrement (∆*T*) between the topside and the underside of the green leaf, temperature increment (∆*T*) of the environment nearby the green leaf before and after the light irradiation with different absorbers.

Apart from local humidity, our absorber engineered leaves can also tune the local temperature. The steady‐state temperatures of the same one leaf in three cases and the coating membranes are plotted in Figure 3c. The leaf is heated much more with the increased temperature (absorbance) of absorbers. The temperature decrement between top and bottom surfaces of the leaf and nearby air surroundings across the transpiration processes is measured as well (Figure 3d). Due to pronounced transpiration manipulation, the temperature decrement between top and bottom surfaces of the leaves becomes much larger with the higher absorbance of absorber and the local ambient temperature increases >3 °C by leaf/PAN but only ≈1 °C by leaf/PAN/CB, respectively, indicating the noticeable local temperature tunability as well. Notably, about six months after the photothermal experiments, the plant engineered with absorbers still keep alive, as supported by the digital images in Figure S5 (Supporting Information).

**Figure** **4**a–c shows the possible heat transfer processes of the absorber–leaf interfaces based tunable transpiration, including light absorption of the leaf, and forward radiative and conductive heat contribution of the absorber to the leaf, backward radiative and convective heat loss of the absorber to the ambient. The weighted solar absorbance (α) of the white or black absorber and the weighted solar absorbance (*α′*) of the green leaf, which are the wavelength‐dependent absorbance (*A*) weighted to the AM 1.5 G solar spectrum can be expressed as shown in Note S1 (Supporting Information).^16^, ^42^, ^43^ For the green leaf engineered with white membrane (Figure 4a), green leaf (Figure 4b), and green leaf coated with black membrane (Figure 4c), the transpiration (about 8.6, 29.3, and 36.4%, respectively) may mainly result from photosynthesis of the green leaf that absorbs the light through translucent white membrane (*α′* = 12.8%) (see Figure S6, Supporting Information) besides the little conductive and radiative heat contribution of the absorber (≈0.1%) and the sublayer green leaf (≈0.4%), direct light absorption of the leaf (α = 54.9%), and large conductive and radiative heat contribution of the absorber (≈90.9%), respectively, as estimated in Note S1 (Supporting Information). The energy input to the leaf is higher than corresponding total consumption of the heat transfer (Figure 4a–c), indicating that there are other auxiliary heat transfer channels for the leaf. It should be noted that the essential difference between our absorber–leaf interface and absorber–water interface is that the infrared thermal conduction and radiation are also helpful for accelerating the plant transpiration.

**Figure 4:**
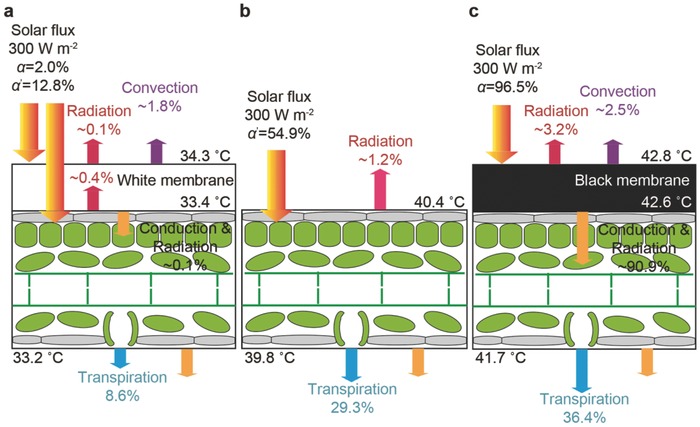
Mechanism of heat transfer in tunable transpiration. Heat transfer diagram of the tunable transpiration of the green plant: a) green leaf coated with white absorber, b) green leaf, and c) green leaf coated with black absorber.

In summary, for the first time we demonstrate that the plant transpiration efficiency could be tuned by engineering the absorber–leaf interfaces. Enhanced light absorption will improve effective thermal contribution of the absorber and transpiration efficiency of the leaf, as well as increases relative humidity and reduces temperature increment of surrounding air of the plant. On the basis of global water cycle, moderate global temperature, humidity, and precipitation may be attainable through the tunable plant transpiration. Moreover, photothermal conversion shows the potential as the regulator of the nature, besides the emerging applications, such as radiative cooling,^42^, ^43^, ^44^, ^45^ thermophotovoltaics,^46^, ^47^, ^48^, ^49^ water purification,^10^, ^11^, ^12^, ^13^, ^14^, ^15^, ^16^, ^17^, ^18^, ^19^, ^20^, ^21^, ^22^, ^23^ seawater desalination,^24^, ^25^, ^26^, ^27^ and power generation.^28^, ^29^

## Experimental Section

*Fabrication of the Membranes*: The green plant was a complete SA, whose plant structure (i.e., roots, stem, and leaf) was well retained. The green plant was fixed to the stainless rotation axle. The PAN solution was obtained by mixing PAN and dimethyl formamide (DMF) with mass ratio of 1:9. The white membrane (PAN membrane) was fabricated by the electrospinning of PAN solution for 1 h onto the top leaf surface, with voltage of 15 kV, feeding rate of 2 mL h^−1^, and distance from needle to the stainless rotation axle of 6 cm. The carbon black (CB) solution was prepared by mixing and ultrasounding CB and methanol solution with the mass volume ratio of 1:100 g mL^−1^. The broadband absorptive black absorber was prepared by the rapid gas blowout of CB solution onto the white membrane with natural drying. It took only about 10 s for the gas blowout to one leaf. The chemical materials were purchased from Sinopharm Chemical Reagent Co., Ltd. (Shanghai, China) and used without further purification.

*Morphological Characterizations*: The morphologies and structures of the samples were characterized by SEM (Dual‐beam FIB 235, FEI Strata). And before identifying the absorber–leaf interface, the samples were frozen at −5 °C for 24 h and sequential frozen dried for 48 h. Note that, after the frozen drying and slicing processes, all the absorber and green leaf may shrink, as shown in Figure 2f,g. In fact, the average thicknesses of the PAN membrane, fresh green leaf, and PAN/CB membrane were measured about 100, 330, and 100 µm using a vernier caliper, respectively.

*Optical Measurements*: Optical transmittance and reflectance measurements on the absorbers were carried out using different optical measurement systems. A Shimadzu UV‐3600 spectrophotometer attached with an integrating sphere (ISR‐3100) was used for the hemispherical reflectance measurements in the UV–vis–NIR range (200 nm to 2.5 µm). A Thermo Nicolet 6700 FT‐IR spectroscope with a Perkin‐Elmer integrating sphere was used for the hemispherical reflectance in the MIR regime (500–4000 cm^−1^, 2.5–20 µm), which was calibrated with a standard Au reference and a resolution of 4 cm^−1^. The absorbance was then calculated by *A* = 1 − *R* − *T*, *R* and *T* are the reflectance and transmittance, respectively.

*Plant Transpiration Measurements*: The experimental setup of the plant transpiration measurement was composed of a solar simulator (Newport 94043A), a double‐lens focusing system (200 mm focal length and 100 mm diameter for lens no. 1, 50 mm focal length and 30 mm diameter for lens no. 2; Beijing Optical Century Instrument Co.), a power meter (an air cool thermopile sensor with a 19 mm diameter detector, 30 W, PM30; No. 1098314, Coherent), a test sample holder, a balance (FA2004, 0.1 mg accuracy), thermocouples, a serial communication module (RS232), a desktop computer, a humidity senor (TH100, Mingle), and a graduated test tube. The experiments were typically conducted at an ambient temperature of 25 °C and humidity of ≈40%. The illumination source was a solar simulator (Class AAA, Newport 94043A) with an optical filter for the standard air mass 1.5‐G spectrum. The output solar irradiation of the solar simulator was focused by the double‐lens system and projected on the surface of the sample. The power density of the illumination was measured at the level of the sample with the power meter. By carefully optimizing the double‐lens focusing system, the maximal irradiation power density to prevent the green leaf from being sunburned was ≈300 W m^−2^ (0.3 sun), and the effective irradiated area of light spot is about ≈6 × 10^−4^ m^2^, which is smaller than that of the green leaf. The designed test sample holder was a plastic foam board to fix the green leaf and to minimize the measurement error of temperature induced by the sample holder. The balance (FA2004, 0.1 mg accuracy) was used to monitor the mass loss from transpiration, recorded in real time by the desktop computer (with RS 232 serial ports), and then used to determine the transpiration rate and transpiration efficiency of plant transpiration. The change of relative humidity of the environment nearby the green leaf was measured using a humidity senor (TH100, Mingle). And water uptake of the green plant for transpiration was measured by the water loss in the graduated test tube. The temperature of the topside of the membrane and the top/bottom surface of the leaf, temperature difference between the top and bottom surfaces of the leaf, temperature change of the environment nearby the leaf before and after the light irradiation were recorded by four thermocouples (placed on top surface of the membrane, top/bottom surface of the green leaf, and in the air 5 cm nearby the green leaf, respectively). All the thermocouples were coated with a highly reflective white titanium oxide coating by atomic layer deposition to suppress the heating effect of direct illumination.

## Conflict of Interest

The authors declare no conflict of interest.

## Supporting information

Supplementary
