# Supplementary material for: Tuning Transpiration by Interfacial Solar Absorber‐Leaf Engineering
Source: Adv Sci (Weinh). 2017 Dec 2;5(2):1700497. doi: 10.1002/advs.201700497 (PMC5827646; doi:10.1002/advs.201700497)
Supplement: Supplementary file 1 — Supplementary [file ADVS-5-1700497-s001.pdf]

Copyright WILEY-VCH Verlag GmbH & Co. KGaA, 69469 Weinheim, Germany, 2017.

Supporting Information

for *Adv. Sci.,* DOI: 10.1002/advs.201700497

Tuning Transpiration by Interfacial Solar Absorber-Leaf Engineering

Shendong Zhuang, Lin Zhou, Weichao Xu, Ning Xu, Xiaozhen Hu, Xiuqiang Li, Guangxin Lv, Qinghui Zheng, Shining Zhu, Zhenlin Wang, and Jia Zhu**

Copyright WILEY-VCH Verlag GmbH & Co. KGaA, 69469 Weinheim, Germany, 2016.

Supporting Information

Title: Tuning transpiration by interfacial solar absorber-leaf engineering

Shendong Zhuang[†]*, Lin Zhou*[†]*, Weichao Xu, Ning Xu, Xiaozhen Hu, Xiuqiang Li, Guangxin Lv, Qinghui Zheng, Shining Zhu, Zhenlin Wang*, Jia Zhu**

[†] These authors contributed equally to this work.

National Laboratory of Solid State Microstructures, College of Engineering and Applied Sciences, School of Physics, and Collaborative Innovation Center of Advanced Microstructures, Nanjing University, Nanjing 210093, P. R. China.

* E-mail: zlwang@nju.edu.cn (Z. Wang); jiazhu@nju.edu.cn (J. Zhu).

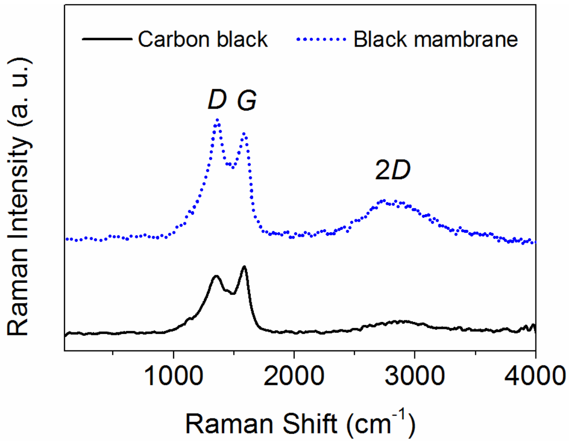

Figure S1. Raman spectra of the membranes.

The Raman spectra of as-prepared black membrane (PAN/CB) and carbon black (CB) are plotted in Figure S1. Both those of the CB and black membrane show three peaks at about 1342, 1586, and 2700 cm^{-1}, corresponding to the *D*, *G*, and 2*D* band of graphite, respectively. The *D* band (breathing mode of *κ*-point phonons with A_{1g} symmetry) is connected with the defects and disorder in the hexagonal lattice and requires defect for activation. The *G* band (*Γ*-point phonons with E_{2g} symmetry at the Brillouin zone center) is arisen from the vibration of sp^2-bonded carbon atoms. The

2D band corresponds to the second order of the D peak. And it originates from a process where momentum conservation is satisfied by two phonons with opposite wave vectors, no defects are required for their activation.[1] Additionally, the Raman signal of the PAN is not be observed.

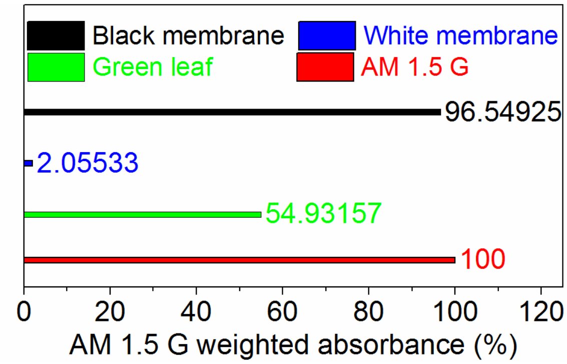

Figure S2. Weighted absorbance of the membranes relative to the air mass 1.5 global tilt solar spectrum (AM 1.5 G).

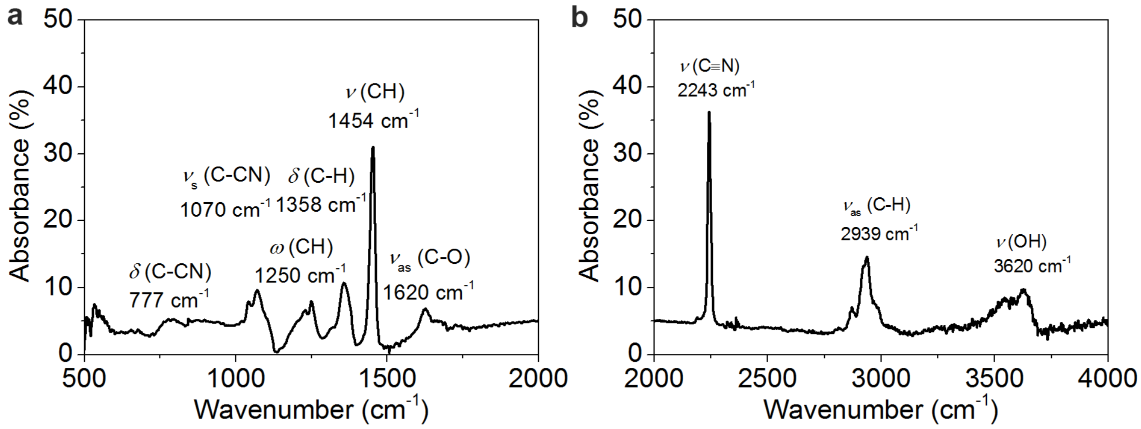

Figure S3. Experimental FT-IR measured by an integrated sphere in the wavenumber regime.

FT-IR of PAN membrane are shown in Figure S3. The bands at 777, 1070, 1250, 1358, 1620, 2939, and 3620 cm^{-1} are signs of vibrations of –C–CN,[2] symmetrical stretching of –C–CN,[3] –CH,[3] –C–H,[2] –C=C in the cyclic structure,[3] –C–H asymmetrical stretching of the –CH_2– groups in the PAN backbone,[3] and –OH stretching that due to the intermolecular hydrogen interactions and water contribution.[2]

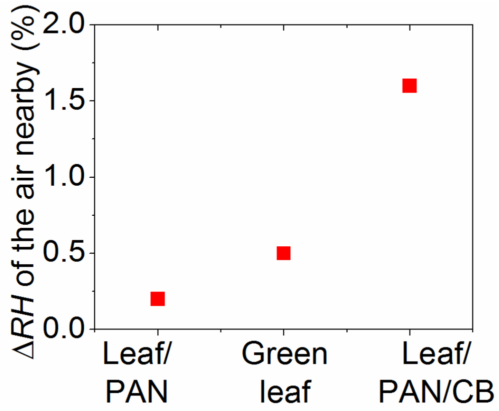

Figure S4. Change of relative humidity (Δ*RH*) of the environment nearby the green leaf engineered with different absorbers before and after 1 hour-illumination under 0.3 sun.

We can anticipate that interfacial engineering of the leaves/absorbers could have impact on relatvie humidity control of local environment. We have clear data showing the humidification of tunable plant transpiration to local environment. The relative humidity (*RH*) increment is about 0.2% for Leaf/PAN, 0.5% for green leaf, and 1.6% for Leaf/PAN/CB, respectively. Corresponding water mass provided by the plants is about 2.28×10^{-5} kg for Leaf/PAN, 7.85×10^{-5} kg for green leaf, and 9.77×10^{-5} kg for Leaf/PAN/CB, respectively, as shown in Table S1 of the Supporting Information. Herein, the effective irradiated area of the leaves is about ~6×10^{-4} m^2, and the humidified volume is about 7.332×10^{-3} m^3 (for the electronic balance of FA2004 with the size of 195 mm × 160 mm × 235 mm). Generally, the water mass of about 0.2 kg (200 mL) per hour is expected to humidify the volume of 300 m^3 of the residential space (i.e. area: 100 m^2, hight: 3 m). Hence, it is resonable that 5.26 m^2, 1.53 m^2, and 1.23 m^2 of the leaves for the Leaf/PAN, Green leaf, and Leaf/PAN is suitable for humidifying the the common residence, respectively. Namely, it corresponds to about 36, 10, and 8 pots of plants of Leaf/PAN, Green leaf, and Leaf/PAN with 100 leaves, respectively. In short, about 8 pots of green plant with the leaves coated by PAN/CB black absorber could humified the common residence via the photo-thermal conversion method.

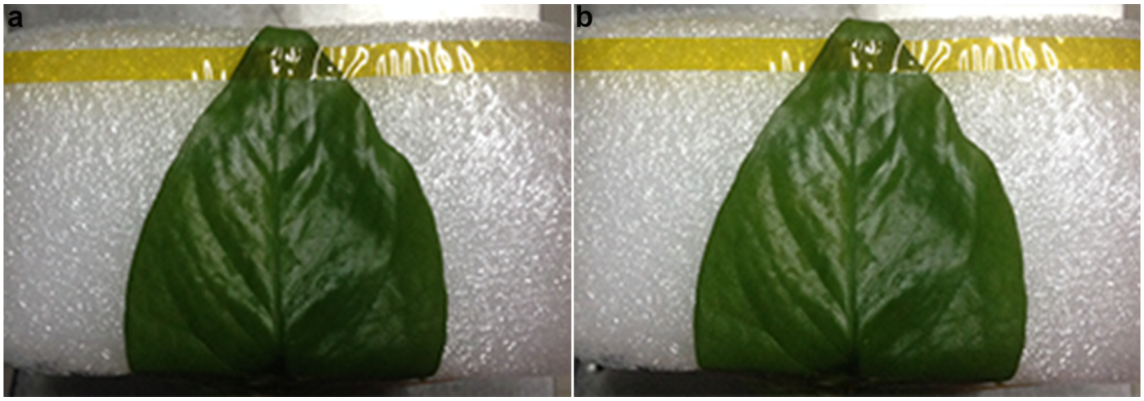

Figure S5. Digital image of the green leaf (a) before and (b) 6 months after the photo-thermal experiments.

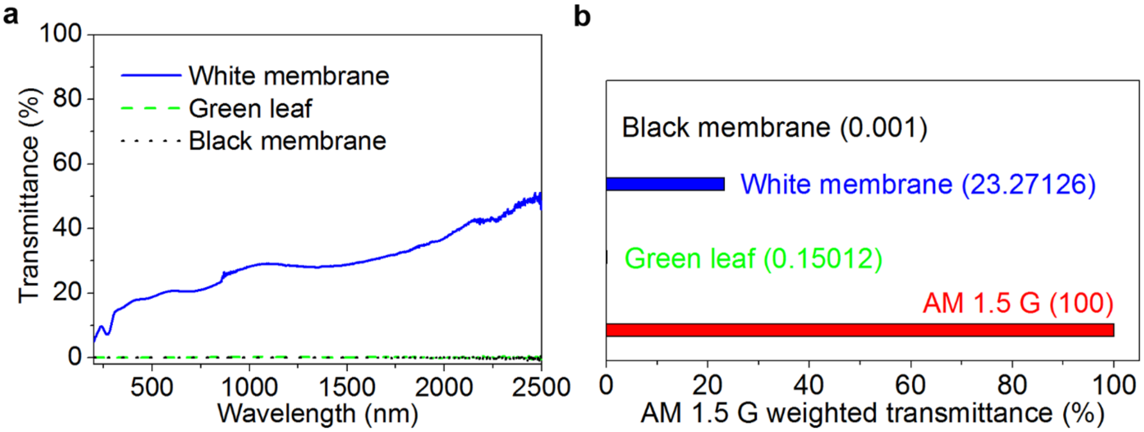

Figure S6. (a) Transmittance spectra and (b) average weighted solar transmittance of the absorbers relative to the air mass 1.5 global tilt solar spectrum (AM 1.5 G).

The weighted absorbance of the leaf (α') to the light through white membrane in the white absorber-leaf system is about ~12.8%, which is calculated based on the average weighted solar transmittance of the white membrane (~23%, Figure S6b, Supporting Information) and the weighted absorbance of the green leaf (~55%, , Figure S2, Supporting Information).

Table S1: Parameter summary of the plant transpiration in this work.

Samples	Mass change (kg) @1h	Evaporation rate (kg m^{-2} h^{-1})	η_{Transp} (%)	$\Delta\eta_{Transp}$ (%)	*T* of membrane (˚C)	*T* of leaf (˚C, top side	*T* of steam (˚C, underside)	Δ*T* of leaf surfaces (˚C)	Δ*T* of air nearby (˚C)	Δ*RH* of air nearby (%)	Water loss (mL)
**Green leaf + White membrane**	2.28×10^{-5}	0.038	8.56	-70.82	34.3	33.4	33.2	-0.2	3.3	0.2	0.02
**Green leaf**	7.85×10^{-5}	0.131	29.32	0	40.4	40.4	39.8	-0.6	1.8	0.5	0.08
**Green leaf + Black membrane**	9.77×10^{-5}	0.164	36.44	24.27	42.8	42.6	41.7	-0.9	1.1	1.6	1.00

Note that Samples refer to the same one green plant cultivated in graduated test tube, which is coated with no membrane, with white membrane, and with black membrane, respectively. Mass change @1h is the mass change of the plant system (green plant + graduated test tube + water in the graduated test tube + membrane) recorded by the electronic balance over 1 hour. η_{Transp} is the plant transpiration efficiency. $\Delta\eta_{Transp}$ is the transpiration efficiency of green plant with membrane relative to the green plant without membrane covering. *T* (temperature, ˚C) of membrane is the

temperature of the topside of the membrane. *T* of leaf is the temperature of the top side of the green leaf. *T* of steam is recorded around the underside of the green leaf, where the steam is escaped from the stoma at the underside of the leaf. Δ*T* of leaf surfaces is the temperature difference between the top side and the underside of the green leaf. Δ*T* of air nearby is the temperature change of the environment nearby the green leaf. Δ*RH* of air nearby is the change of relative humidity of the environment nearby the green leaf. Water loss is the volume loss of the water in the graduated test tubes.

Table S2. Summary of the plant transpiration efficiency in the previous reports.

Samples	Transpiration rate (kg m^{-2} h^{-1})	Transpiration efficiency (%)
Pelargonium hortorum[4]	0.01	0.06796
Vicia faba[4]	0.03	0.20387
Coriander[5]	0.01781	1.20786
Tobacco[6]	0.042	2.83465
Vicia faba[4]	0.00045	3.0581
Avocado[7]	0.04536	3.07629
Tobacco[6]	0.048	3.2396
Tobacco[6]	0.049	3.30709
Pelargonium hortorum[4]	0.5	3.39788
Tobacco[6]	0.053	3.57706
Tobacco[6]	0.059	3.98201
Tobacco[6]	0.067	4.52194
Avocado[7]	0.07128	4.83417
Cotton-Carolina Queen[8]	0.07775	5.27296
Cotton-Empire[8]	0.08725	5.91725
Berseem clover[9]	0.1296	8.7894
Cassava[10]	0.14969	10.06459
Cotton-Auburn[8]	0.17575	11.91927

Cotton-Smooth Leaf Empire[8]	0.17875	12.12273
Cotton[10]	0.198	13.36335
Cassava[10]	0.20412	13.72444
Xanthium strumarium L.[11]	0.21	14.2811
Soybean-Beinville[8]	0.22075	14.97114
Soybean-Hardee[8]	0.2225	15.08983
Cotton[12]	0.225	15.18563
Cassava[10]	0.23134	15.57795
Tomatoes-Marion[8]	0.23225	15.75107
Tomatoes-Rutgers[8]	0.23375	15.8528
Cassava[10]	0.23911	16.05282
Soybean-Hampton[8]	0.25675	17.41264
Tomatoes-Marglobe[8]	0.26075	17.68392
Sorghum-Amak-R-12[8]	0.275	18.65035
Sorghum-NK-210[8]	0.2875	19.49809
Sorghum-RS-610[8]	0.29375	19.92196
Corn-MP 305×MP 307[8]	0.29625	20.09151
Corn-MP 305×T 101[8]	0.31425	21.31226
Corn-MP 399×MP 311[8]	0.3225	21.87177
Corn-Dixie 82[8]	0.361	24.48282
Berseem clover[9]	0.40824	27.68661
Scindapsus aureus coated with white PAN membrane[this work]	0.03824	8.55564
Scindapsus aureus[this work]	0.13164	29.3197
Scindapsus aureus coated with black PAN/CB membrane[this work]	0.16384	36.4352

Note S1.

Heat transfer for the tunable transpiration of green leaf in the case of (a) green leaf coated with white membrane, (b) green leaf, and (c) green leaf coated with black membrane can be respectively expressed as follows:[13]

(a) Green leaf coated with white membrane:

For the absorber:

$$\alpha = \eta_{Rad} + \eta_{Conv} + \eta_{Cond} = 2.05533\% \tag{1}$$

$$\begin{aligned}\eta_{Rad} &= \frac{q_{Rad}}{q_{solar}} = \frac{\varepsilon\sigma(T^4 - T_\infty^4)}{q_{solar}} \\ &= \frac{0.04579 \times 5.670367 \times 10^{-8}\ W\ m^{-2}\ K^{-2} \times (307.45^4 - 306.35^4) K^4}{300\ W\ m^{-2}} \\ &= \frac{0.33024\ W\ m^{-2}}{300\ W\ m^{-2}} \\ &= 0.11008\%\end{aligned} \tag{2}$$

$$\begin{aligned}\eta_{Conv} &= \frac{q_{Conv}}{q_{solar}} = \frac{h(T - T_\infty)}{q_{solar}} \\ &= \frac{5\ W\ m^{-2}\ K^{-1} \times (307.45 - 306.35) K}{300\ W\ m^{-2}} \\ &= \frac{5.5\ W\ m^{-2}}{300\ W\ m^{-2}} \\ &= 1.83333\%\end{aligned} \tag{3}$$

$$\eta_{Cond} = \alpha - \eta_{Rad} - \eta_{Conv} = 0.11192\% \tag{4}$$

For the sublayer green leaf:

$$\alpha' = 12.7833\% \tag{5}$$

$$\eta'_{Rad} = \frac{q'_{Rad}}{q_{solar}} = \frac{\varepsilon\sigma(T'^4 - T'^4_\infty)}{q_{solar}}$$

$$= \frac{0.85802 \times 5.670367 \times 10^{-8}\ W\ m^{-2}\ K^{-2} \times (306.55^4 - 306.35^4)K^4}{300\ W\ m^{-2}} \quad (6)$$

$$= \frac{1.12015 W\ m^{-2}}{300\ W\ m^{-2}}$$

$$= 0.37338\%$$

$$\eta_{Transp} = 8.55564\% \quad (7)$$

$$\eta_{Cond} + \alpha' = \eta_{Transp} + \eta'_{Rad} + \dots\ \dots = 12.89522\% \quad (8)$$

Due to the white absorber significantly reduces the thermal contribution to the leaf and suppresses the plant transpiration, the transpiration of the green leaf in this case ($\eta_{Transp} = 8.55564\%$) may result from the photosynthesis induced by the absorption of the leaf to the light through translucent white absorber (α'=12.8%) (Figure S6, Supporting Information). The conductive heat contribution (η_{Cond}) of white absorber to green leaf should be ~0.1%, based on $\eta_{Cond} = \alpha - \eta_{Rad} - \eta_{Conv}$. Apart from making contribution to the leaf transpiration, the transmitted light may also provide backward radiative heat (η'_{Rad}) to the upper-layered white absorber.

(b) Green leaf without absorber engineering:

$$\alpha' = \eta_{Transp} + \eta'_{Rad} + \dots\ \dots = 54.9315\% \quad (9)$$

$$\eta_{Transp} = 29.3197\% \quad (10)$$

$$\eta_{Rad} = \frac{q_{Rad}}{q_{solar}} = \frac{\varepsilon\sigma(T^4 - T^4_\infty)}{q_{solar}}$$

$$= \frac{0.85802 \times 5.670367 \times 10^{-8}\ W\ m^{-2}\ K^{-2} \times (313.55^4 - 312.95^4)K^4}{300\ W\ m^{-2}} \quad (11)$$

$$= \frac{3.58917\ W\ m^{-2}}{300\ W\ m^{-2}}$$

$$= 1.19639\%$$

(c) Green leaf coated with black membrane:

For the black absorber:

$$\alpha = \eta_{Rad} + \eta_{Conv} + \eta_{Cond} = 96.54925\% \tag{12}$$

$$\begin{aligned}
\eta_{Rad} &= \frac{q_{Rad}}{q_{solar}} = \frac{\varepsilon\sigma(T^4 - T_\infty^4)}{q_{solar}} \\
&= \frac{0.9098 \times 5.670367 \times 10^{-8}\ W\ m^{-2}\ K^{-2} \times (315.95^4 - 314.45^4)K^4}{300\ W\ m^{-2}} \\
&= \frac{9.69325\ W\ m^{-2}}{300\ W\ m^{-2}} \\
&= 3.23108\%
\end{aligned} \tag{13}$$

$$\begin{aligned}
\eta_{Conv} &= \frac{q_{Conv}}{q_{solar}} = \frac{h(T - T_\infty)}{q_{solar}} \\
&= \frac{5\ W\ m^{-2}\ K^{-1} \times (315.95 - 314.45)K}{300\ W\ m^{-2}} \\
&= \frac{7.5\ W\ m^{-2}}{300\ W\ m^{-2}} \\
&= 2.5\%
\end{aligned} \tag{14}$$

$$\begin{aligned}
\eta_{Cond} &= \frac{q_{Cond}}{q_{solar}} = \frac{q_{leaf}}{q_{solar}} = -\frac{k_{leaf}\dfrac{\Delta T_{leaf}}{L}}{q_{solar}} \\
&= \frac{0.1\ W\ m^{-1}\ K^{-1} \times \dfrac{-(314.85 - 315.75)K}{3.3 \times 10^{-4}\ m}}{300\ W\ m^{-2}} \\
&= \frac{272.72727\ W\ m^{-2}}{300\ W\ m^{-2}} \\
&= 90.90909\%
\end{aligned} \tag{15}$$

For the sublayer green leaf:

$$\eta_{Transp} = 36.4352\% \tag{16}$$

Herein, η_{Transp}, η_{Rad}, η_{Conv}, and η_{Cond} are the transpiration efficiency of the green leaf, and radiation, convection, and conduction efficiency of the membrane, respectively. $\dot{m}$ is the

transpiration rate, h_{LV} is the latent heat, q_{solar} is the actual illumination intensity (300 W m^{-2}). q_{Rad}, q_{Conv}, and q_{Cond} are the radiative, convective, and conductive heat flux of the membrane, respectively. ε is the emittance of the absorbers (about 0.04579 for white membrane, 0.85802 for green leaf and 0.9098 for black membrane), which are determined by the reflectance with the blackbody emission spectrum at corresponding temperatures of absorbing surfaces. σ is the Stefan-Boltzmann constant (5.670367×10^{-8} W m^{-2} K^{-4}), and h is the convection heat transfer coefficient (5 W m^{-2} K^{-1}).[14] q_{leaf} is the heat flux from the top surface to the bottom surface of the leaf, which can be expressed as follows based on the Fourier's law:[15]

$$q_{leaf} = -k_{leaf} \frac{\Delta T_{leaf}}{L} \tag{17}$$

where k_{leaf} is the thermal conductivity of the green leaf, which is about 0.1 W m^{-1} K^{-1},[16,17] ΔT_{leaf} the temperature difference between the top surface and the bottom surface of the leaf, and L the measured thickness of the leaf (about 330 μm, 3.3×10^{-4} m).

References

[1] C. Casiraghi, S. Pisana, K. S. Novoselov, A. K. Geim, A. C. Ferrari, *Appl. Phys. Lett.* **2007**, *91*, 233108.

[2] E. V. Loginova, I. V. Mikheev, D. S. Volkov, M. A. Proskurnin, *Anal. Methods* **2016**, *8*, 371.

[3] J. Zhao, J. Zhang, T. Zhou, X. Liu, Q. Yuan, A. Zhang, *RSC Adv.* **2016**, *6*, 4397.

[4] D. F. Parkhurst, D. M. Gates, *Nature* **1966**, *210*, 172.

[5] M. Kerton, H. J. Newbury, D. Hand, J. Pritchard, *J. Exp. Bot.* **2009**, *60*, 227.

[6] I. Zelitch, *Science* **1964**, *143*, 692.

[7] M. M. Blanke, C. J. Lovatt, *Ann. Bot.* **1993**, *71*, 543.

[8] J. E. Pallas, *Science* **1965**, *147*, 171.

[9] M. Lazaridou, S. D. Koutroubas, Drought effect on water use efficiency of berseem clover at various growth stages, presented at *4th International Crop Science Congress*, Brisbane, Queensland, Australia, September - October, **2004**.

[10] I. Aspiazú, T. Sediyama, J. I. Ribeiro Jr, A. A. Silva, G. Concenco, E. A. Ferreira, L. Galon, A. F. Silva, E. T. Borges, W. F. Araujo, *Planta Daninha* **2010**, *28*, 699.

[11] K. Raschke, *Planta* **1975**, *125*, 243.

[12] L. T. Liu, Y. Zhang, J. Jiao, H. J. Lu, *Acta Chim. Sinica* **2013**, *71*, 535.

[13] G. Ni, G. Li, Svetlana V. Boriskina, H. Li, W. Yang, T. Zhang, G. Chen, *Nature Energy* **2016**, *1*, 16126.

[14] X. Li, W. Xu, M. Tang, L. Zhou, B. Zhu, S. Zhu, J. Zhu, *Proc. Nat. Acad. Sci.* **2016**, *113*, 13953.

[15] D. Manzano, M. Tiersch, A. Asadian, H. J. Briegel, *Phys. Rev. E* **2012**, *86*, 061118.

[16] Y. S. Touloukian, R. W. Powell, C. Y. Ho, P. G. Klemens, *Thermophysical Properties of Matter-The TPRC Data Series. Vol 2. Thermal Conductivity-Nonmetallic Solids*, Plenum Publishing Corporation, New York, NY, USA **1971**.

[17] Wikipedia, List of thermal conductivities, https://en.wikipedia.org/wiki/List_of_ther-mal_conductivities, accessed: December, **2016**.
